# Pneumococcal Infection and Vaccination-Related Knowledge, Attitudes and Practices Among Saudi Residents

**DOI:** 10.3390/pathogens14070711

**Published:** 2025-07-18

**Authors:** Wed S. Althobaiti, Abeer D. Alnefaie, Kaifah M. Althaali, Ola M. Alsufyani, Yassmin M. Shebany, Ayman A. Atalla, Ibtihal M. Alotaibi, Eman Y. Santali, Sayed F. Abdelwahab

**Affiliations:** 1 College of Pharmacy, Taif University, Taif 21944, Saudi Arabia; s44010045@students.tu.edu.sa (W.S.A.); s44005726@students.tu.edu.sa (A.D.A.); s44003609@students.tu.edu.sa (K.M.A.); s43802295@students.tu.edu.sa (O.M.A.); s44010023@students.tu.edu.sa (I.M.A.); 2 Biology Department, Faculty of Science, Taif University, Taif 21944, Saudi Arabia; m.yassmin@tu.edu.sa; 3 Family Medicine Department, College of Medicine, Taif University, Taif 21944, Saudi Arabia; dr.ayman@tu.edu.sa; 4 Pharmaceutical Chemistry Department, College of Pharmacy, Taif University, Taif 21944, Saudi Arabia; eysantali@tu.edu.sa; 5 Pharmaceutics and Industrial Pharmacy Department, College of Pharmacy, Taif University, Taif 21944, Saudi Arabia

**Keywords:** attitudes, knowledge, pneumococcal conjugate vaccines, pneumococcal infection, practice, Saudi Arabia, vaccines

## Abstract

**Background and aims**: Pneumococcal infections, primarily caused by *Streptococcus pneumoniae*, pose significant global public health challenges, particularly in vulnerable populations. In Saudi Arabia, the introduction of pneumococcal vaccination has been a crucial step towards its prevention. However, gaps in public knowledge and attitudes toward the vaccine may hinder its effectiveness. Recent studies indicate a lack of awareness about the benefits of pneumococcal vaccination, suggesting a need for further investigation. This study determined the knowledge, attitudes, and practices (KAP) of Saudi residents regarding pneumococcal infection and vaccination. **Study design and Methods**: This observational cross-sectional study was conducted across Saudi Arabia from November 2024 to March 2025. Data were collected through a self-administered online questionnaire designed to evaluate KAP towards pneumococcal infection and vaccination. The sample size was calculated to be 385 participants. The questionnaire underwent expert validation and a pilot study to ensure clarity and reliability. The questionnaire was distributed via social media platforms to collect the data. Data management was conducted using Microsoft Excel, and statistical analysis was performed using Statistical Package for Social Sciences (SPSS) software version 26. **Results**: This study included 1230 participants of whom 630 (51.2%) were females and 1075 (87.4%) were Saudi citizens. Almost half of the participants (50.2%) were aged 18–30 years, and 498 (40.5%) were married. The average knowledge score was 58.6%, indicating a moderate level of understanding among the participants regarding pneumococcal infection. Also, the average attitudes score was 70.6%, reflecting a generally positive outlook towards the importance of pneumococcal vaccination and its potential to limit infection spread. In addition, the average practices score was 68%, indicating a fairly good level of behaviors regarding vaccination practices. Statistical analyses showed that demographic factors and clinical characteristics significantly shape individuals’ KAP towards pneumococcal infection and vaccination. **Conclusions:** This study highlights the critical need to improve KAP regarding pneumococcal infections and vaccinations among Saudi residents and could help in developing more targeted and effective public health interventions to protect Saudi residents from pneumococcal infections.

## 1. Introduction

Pneumococcal infection is primarily caused by *Streptococcus pneumoniae* (*S. pneumoniae*), which is an encapsulated bacterium with a polysaccharide capsule, an essential factor in its virulence. Pneumococcal infection is transmitted by direct contact with respiratory secretions from patients and healthy carriers. Serious pneumococcal infections include pneumonia, meningitis, bacteremia, and otitis media [1]. Pneumococci are major causes of morbidity and mortality worldwide. High-risk individuals for pneumococcal pneumonia include young children (younger than five years), the elderly (65 years or older), pregnant women, and those with compromised immune systems or specific chronic medical conditions, such as those with diabetes mellitus, chronic heart diseases, kidney diseases, liver diseases, lung diseases, or cigarette smokers [2]. While pneumococcal vaccination is a proven preventive measure, its success in reducing the burden of the disease depends not only on its availability but also on the public’s understanding and willingness to engage in preventive behaviors. In Saudi Arabia, several million pilgrims from around the globe arrive in Mecca yearly for the Hajj and/or Umrah, presenting a major public health challenge in terms of infectious disease transmission, including pneumococcal infections [3]. Also, due to the varying levels of health literacy, it is vital to evaluate the public’s knowledge, attitudes, and practices (KAP) towards pneumococcal infection and its vaccination [4].

Pneumococcal conjugate vaccines (PCVs) come in three primary types: the 7-valent (PCV7), 10-valent (PCV10), and 13-valent (PCV13) vaccines. There is also a fourth type of pneumococcal non-conjugate vaccine, the 23-valent pneumococcal polysaccharide vaccine (PPSV23) [5]. Active immunization is the main use of PCVs, particularly in newborns, young children, and populations with specific conditions such as those listed above [6,7]. The four doses of PCV that are normally administered at 2, 4, 6, and 12–15 months of age provide long-term protection against pneumococcal infection [8]. With its 23 different types of pneumococcal coverage, PPSV23 provides broader protection and is advised for high-risk individuals under and over 65 years of age [3,9]. Long-lasting immunity is provided as well by PCV13; nevertheless, high-risk patients may require booster doses. After approximately five years, a booster dose is recommended for high-risk individuals using PPSV23 [6]. Importantly, regardless of the global and international efforts to promote vaccination, prior studies have revealed important gaps in public awareness and mistaken beliefs about pneumococcal diseases. A recent study pointed out that although there is awareness of the pneumococcal vaccine, only 17% of the participants reported being informed about its benefits, suggesting a significant knowledge deficit [10]. Also, research has shown that false information about the disease and its vaccine contributes to decreasing acceptance rates, especially among high-risk groups [10]. These gaps can cause low vaccine uptake, preventing the achievement of optimal coverage rates, leaving a large part of the population susceptible to a preventable infection. In addition, the Saudi Thoracic Society guidelines emphasized the need for targeted health education campaigns and enhancement of public health strategies to promote knowledge and encourage positive health-seeking behaviors related to pneumococcal vaccination among the Saudi population [11].

In Saudi Arabia, the estimated incidence of invasive pneumococcal disease in children under the age of five examined in the period between 2007 and 2009 was 2.5–21.6 cases per 100,000, indicating a huge health concern in the country [12]. The introduction of the pneumococcal vaccine in the national immunization program in 2009 [13] has been a positive step, but the actual effect on public health is dependent on the population’s KAP toward both the disease and its prevention, which may hinder the vaccine’s effectiveness. In this regard, the Saudi Ministry of Health reports show that the annual pneumococcal vaccination coverage among Saudi children (reported between 2014 and 2018) hovered around 98%. However, very low vaccination rates were found among adults at risk of pneumonia, reaching only 12.9% [14]. Understanding the sociodemographic factors that form health behaviors is necessary for developing specific strategies to increase vaccine acceptance and public engagement [4,15]. Recent studies indicate a lack of awareness about the benefits of pneumococcal vaccination, suggesting a need for further investigation. Therefore, this study examined the KAP towards pneumococcal infection and vaccination among residents of Saudi Arabia. This study offered crucial knowledge about factors influencing vaccine hesitancy and acceptance by examining the degree of public understanding and recognizing misconceptions. Also, demographic factors such as age, education, and socioeconomic status were investigated to see how they influence KAP, giving an overall analysis of the challenges and opportunities to broaden the coverage of pneumococcal vaccine in Saudi Arabia. The findings of this study could help in developing more targeted and effective public health interventions to protect Saudi residents from pneumococcal infections.

## 2. Methods

### 2.1. Study Design

This was an observational cross-sectional study that was conducted in Saudi Arabia from November 2024 to March 2025 to measure KAP related to *S. pneumoniae* (pneumococcal) infection and its vaccination among Saudi residents.

### 2.2. Sample Size Calculation

The sample size was calculated by the sample size equation: n = z^2^ × p (1 − p)/e^2^ where: “n” is the sample size; “z” (1.96) is the z-score for a level of confidence of 95% according to the standard normal distribution; “p” is the sample proportion, expressed as decimal, and “e” (0.05) is the margin of error. The calculated sample size was 385 participants.

### 2.3. Study Tool and Measures

The study used a simple random sampling technique, and a structured self-administered online questionnaire was designed after consulting previously published literature [9,15,16,17,18,19]. The questionnaire consisted of the following four sections:

*Section 1: Demographic questions:* This section contained fifteen questions that asked the participants about their sociodemographic data to provide information about their gender, age, residence, educational level, income, number of children, pneumococcal vaccination status, and other demographic and clinical characteristics.

*Section 2: Knowledge questions:* This section consisted of twelve questions that assessed the participants’ knowledge of pneumococcal infection and its vaccination, e.g., Cause of pneumococcal infection, its complications, high-risk individuals for S. pneumoniae infection, and number of pneumococcal vaccine protective doses, etc.

*Section 3: Attitude questions:* This section consisted of nine questions that measured the participant’s attitude towards S. pneumonia infection and its vaccination.

*Section 4: Practice questions:* This last section contained nine questions that measured the participants’ practices towards pneumococcal infection and its vaccination.

### 2.4. Validation and Reliability of the Questionnaire

The questionnaire was examined by a group of four experts from Taif University’s College of Pharmacy for face and content validity. They were asked to assess the regional settings’ clarity, consistency, and suitability. Their suggestions were incorporated into the final version of the questionnaire. A pilot sample of 32 volunteers was also used in a field test to validate the questionnaire. The average time that the participants took to complete the questionnaire in the pilot study was 6–8 min, and their data were excluded from the study. Although all variables were intended to be analyzed individually, we checked the reliability of the items assessing residents’ pneumococcal-related KAP using the pilot sample data and we obtained a Cronbach’s alpha value of ≥0.7.

### 2.5. Study Settings and Participants

The study was performed among Saudi residents aged 18 years and older in all Saudi Arabian regions. A self-administered online survey was used to collect the data using a random sampling technique. The study population included individuals aged 18 years and above living in various regions of Saudi Arabia. Participants were selected using a random sampling method to ensure a representative sample. The inclusion criteria include individuals of all genders, residents of Saudi Arabia (whether citizens or foreign residents of the country), those willing to participate, and give consent for participation and able to complete the self-administered questionnaire. Exclusion criteria include individuals under 18 years of age, non-residents of Saudi Arabia, and those who decline to provide online consent for participation. The study aimed to capture a diverse sample to assess the Saudi population’s KAP regarding pneumococcal infection and its vaccination.

### 2.6. Ethical Approval

The study received ethical approval from the Ethics Committee at Taif University under approval number (46-075) prior to its commencement.

### 2.7. Statistical Analysis

Statistical analysis was performed using the Statistical Package for Social Sciences (SPSS) software, version 26.0 (IBM, New York, NY, USA). Descriptive statistics were generated for the responses and their frequencies were presented as numbers and percentages. Correlation coefficients were used to describe the relationships between continuous variables. For independent variables, the Chi-square test was used to compare categorical variables. A *p*-value of <0.05 was considered significant [20,21,22].

## 3. Results

Table 1 shows the demographic characteristics of the study participants. The participants were almost equally distributed regarding sex, with females accounting for 51.2% (*n* = 630). Also, young adults at the age of 18–30 years represented almost one half of the participants (*n* = 617; 50.2%). Most of the participants were Saudi citizens (87.4%), with the Western region being the most represented (*n* = 689; 56.0%). As for marital status, approximately a little less than half of the participants (46.3%) were single and 40.5% (*n* = 498) were married. Regarding educational level, most of the participants had a bachelor’s degree (58.5%). Regarding employment status, 50.7% of the participants were either unemployed students or worked in private businesses, and more than half of the participants (56.3%) were making <5000 SAR as their monthly income. Health awareness of the participants is considered high, as 77.2% have heard of pneumonia, with social media (59.5%) being the main source of information. Child-wise, nearly half of the participants (49.7%) have no children, with the remaining half having various numbers of children. Particularly, 9.8% of the participants in the age group 18–30 years were married with one child or more, compared to at least 83% among the remaining older age groups. Also, we have only 38 participants (3.1%) who were 61 years or older (Table 1).

Table 2 shows the clinical characteristics of the study participants. Most of the participants are non-smokers (*n* = 920; 74.8%), and most of them did not have any chronic illness (*n* = 880; 71.5%). Approximately three-quarters of the participants (*n* = 942; 76.6%) did not have respiratory diseases, and more than 85% of them did not have chronic heart disease (*n* = 1054; 85.7). As for the pneumococcal vaccine, 38.3% (*n* = 477) had not received it, and 40.5% (*n* = 498) did not remember their vaccination status, with the remaining participants (only 20.7%, *n* = 255) reporting that they received the vaccine. Also, for those with children, a prominent part (*n* = 377; 30.7%) declared that it was not applicable to them, whereas 29.9% (*n* = 368) informed that their children had not received the pneumococcal vaccine. The remaining clinical characteristics of the participants can be found in Table 2.

Table 3 and Supplemental Table S1 provide a comprehensive overview of participants’ knowledge regarding pneumococcal infection, including cause, mode of transmission, symptoms, high-risk groups, prevention, complications, and vaccination. These were analyzed alongside demographic and clinical characteristics.

Regarding the cause of pneumococcal infection, less than half of the participants reported that bacteria (*n* = 487; 39.6%) are the cause, followed by viruses (*n* = 302; 24.6%) and fungi (*n* = 57; 4.6%). A considerable portion of them (*n* = 384; 31.2%) reported that they do not know. This may reflect a broader misconception about the causes of pneumococcal infections. These data showed statistical associations with many demographic and clinical characteristics (all *p* < 0.001; Table 3 and Supplemental Table S1).

Regarding the mode of transmission of pneumococcal infection, high awareness was found among the participants, where coughing/sneezing was identified as a transmission method by most of them (*n* = 1041; 84.6%). Other transmission routes, like contaminated food/water and direct contact, showed mixed awareness levels, with almost half of the participants being unaware if these were transmission methods of pneumococcal infection (Table 3). This was significantly associated with many demographic and clinical characteristics (*p* < 0.05; Table 3 and Supplemental Table S1).

Regarding symptoms of pneumococcal infection, fever (*n* = 958; 77.9%), cough (*n* = 827; 67.2%), and rapid breathing (*n* = 812; 66.0%) were recognized as common symptoms. In comparison, vomiting (*n* = 605; 49.2%) was less identified, suggesting higher awareness of the symptoms of pneumococcal infection. These responses were significantly associated with many demographic and clinical characteristics (*p* < 0.05; Table 3 and Supplemental Table S1).

Regarding at-risk groups of pneumococcal infection, infants and young children (*n* = 970; 78.9%) were recognized as a high-risk group, followed by those with weakened immune systems (*n* = 951; 77.3%) and adults over 65 years (*n* = 782; 63.6%). Lower recognition for pregnant women (*n* = 722; 58.7%) and adults < 65 years (*n* = 429; 34.9%) was found, suggesting higher awareness of pneumococcal infection risk groups (Table 3). These responses were significantly associated with many demographic and clinical characteristics (*p* < 0.05; Table 3 and Supplemental Table S1).

Regarding the perception of the seriousness of pneumococcal infection, almost half of the participants (*n* = 639; 52.0%) acknowledged the serious complications resulting from pneumococcal infections, while others either disagreed or were uncertain (Table 3). Awareness of pneumococcal infection complications was significantly associated with many demographic and clinical characteristics. Importantly, there was a significant correlation between the participants’ perceived seriousness of pneumococcal disease and their actual vaccination status, where 56.1% of those who think that pneumococci cause serious illnesses received the pneumococcal vaccine compared to only 49.9% among those who do not with a significant proportion of them not remembering if they took the vaccine or not (~40%) (*p* < 0.05; Table 3 and Supplemental Table S1).

Regarding prevention methods of pneumococcal infection, vaccination was noted as a key preventive measure (*n* = 1087; 88.4%), as was covering the mouth and nose when coughing or sneezing (*n* = 863; 70.2%). On the other hand, awareness of practices like avoiding crowded places and proper hygienic measures varied widely (Table 3). This was significantly associated with many demographic and clinical characteristics (*p* < 0.05; Table 3 and Supplemental Table S1).

Regarding the cure of pneumococcal infection, only half of the participants (*n* = 628; 51.1%) were certain about its curability, reflecting a need for better education (Table 3). This was significantly associated with many demographic and clinical characteristics (*p* < 0.05; Table 3 and Supplemental Table S1).

Regarding vaccination awareness, although knowledge of available vaccines is moderate among the participants (*n* = 637; 51.8%), awareness of vaccines’ inclusion in the national immunization program was below average (*n* = 547; 44.5%). This was significantly associated with many demographic and clinical characteristics (*p* < 0.05; Table 3 and Supplemental Table S1).

Regarding pneumococcal vaccine doses and effectiveness, only a quarter of the participants (*n* = 312; 25.4%) correctly knew that four doses were required for complete protection, and this was strongly associated with many demographic and clinical characteristics (*p* < 0.05). Also, vaccine effectiveness was believed by almost three-quarters of the participants (*n* = 909; 73.9%). This was significantly associated with many demographic and clinical characteristics (*p* < 0.05; Table 3 and Supplemental Table S1).

Awareness of the relationship between pneumococcal and influenza vaccines was low, with only a quarter of the participants (*n* = 309; 25.1%) knowing that they are different. This was significantly associated with many demographic and clinical characteristics. It should be noted that participants with higher socioeconomic status were more likely to recognize the importance of vaccines and be aware of the differences between pneumococcal and influenza vaccines (*p* < 0.05, Table 3 and Supplemental Table S1).

The average knowledge score across the participants was calculated by taking the average percentage of correct answers provided by the participants for the knowledge questions. The average knowledge score was 58.6, indicating that while a foundational understanding exists, significant knowledge gaps remain, necessitating potential educational interventions. Statistically significant differences observed across most knowledge questions indicate strong correlations between knowledge scores and demographic factors such as age, gender, and education, as well as clinical characteristics including smoking status, presence of chronic illnesses, and vaccination history. In this regard, young age participants (18–30 years old) generally had a higher knowledge score compared to other age groups. Also, females tended to have higher knowledge when compared to males in several issues, e.g., transmission routes, risk groups, vaccine availability and its power to prevent pneumococcal infection. Areas lacking significant differences include demographic factors such as lower education levels or socioeconomic status, suggesting targeted education efforts may be beneficial. These findings underscore the influence of both sociodemographic and health-related factors on participants’ awareness and understanding of pneumococcal infection and its vaccination.

Table 4 and Supplemental Table S2 show a detailed distribution of responses to various attitude questions related to pneumococcal infection and vaccination, along with statistics correlating these responses with demographic and clinical characteristics, respectively. In this regard, a total of 597 (48.5%) of the participants strongly agreed/agreed that pneumococcal infection is a serious health threat, while only 317 (25.8%) strongly disagreed/disagreed on this issue. This was significantly associated with many demographic and clinical characteristics (*p* < 0.05). Most of the participants (748; 60.8%) strongly agreed/agreed that it is important for at-risk individuals to receive the pneumococcal vaccine, while only 205 (16.7%) strongly disagreed/disagreed. This was significantly associated with many demographic and clinical characteristics (*p* < 0.05). Also, most participants, 745 (60.6%), strongly agreed/agreed that the pneumococcal vaccine is effective in preventing pneumonia, while only 160 (13%) strongly disagreed/disagreed. This was significantly associated with many demographic and clinical characteristics (*p* < 0.05). In addition, most of the participants, 782 (63.5%), strongly agreed/agreed that pneumococcal vaccination can help limit the spread of infection, while only 178 (14.5%) strongly disagreed/disagreed. This was significantly associated with many demographic and clinical characteristics (*p* < 0.05). Moreover, 61.1% (*n* = 752) of the participants strongly agreed/agreed that they must recommend the pneumococcal vaccine to their family and friends, while only 13.8% (*n* = 170) strongly disagreed/disagreed. This was significantly associated with many demographic and clinical characteristics (*p* < 0.05). Furthermore, most of the participants (*n* = 810; 65.9%) strongly agreed/agreed that it is important for healthcare workers to receive the pneumococcal vaccine, while only 156 (12.7%) strongly disagreed/disagreed. This was significantly associated with many demographic and clinical characteristics (*p* < 0.05; Table 4 and Supplemental Table S2).

Concerns about the possible side effects of pneumococcal vaccine were also reported, with almost half of the participants (*n* = 608; 49.4%) strongly agreeing/agreeing that they are concerned about side effects, while only 226 (18.4%) strongly disagreed/disagreed. This was significantly associated with many demographic and clinical characteristics (*p* < 0.05). Importantly, trust in healthcare professionals was evident as 793 (64.5%) strongly agreed/agreed that they follow medical advice when recommended to take the pneumococcal vaccine, while only 189 (15.4%) strongly disagreed/disagreed. This was significantly associated with many demographic and clinical characteristics (*p* < 0.05). A little more than a third (*n* = 432, 35.1%) of the participants strongly agreed/agreed that their families were healthy and did not need pneumococcal vaccination, while another third (*n* = 397, 32.2%) strongly disagreed/disagreed. This was significantly associated with many demographic and clinical characteristics (*p* < 0.05; Table 4 and Supplemental Table S2).

The average/overall attitude score was calculated by taking the average attitude of each participant (the correct attitude being given a score of 5 and the improper attitude given 1). The score was multiplied by 20 to calculate the percentage. The average/overall attitude score was 70.6, suggesting an overall positive perception toward pneumococcal vaccination. All individual attitude statement scores were sixty or higher, indicating consistently favorable attitudes across the questions. These scores demonstrate agreement among participants regarding the seriousness of pneumococcal infection, the importance of vaccination for at-risk populations, and trust in the vaccine’s effectiveness and healthcare professional recommendations. Statistically significant differences were observed across several attitude items in relation to demographic factors and clinical factors. These findings suggest that while all subgroups show positive attitudes regarding pneumococcal vaccination, continued health education and awareness efforts are still necessary to maintain and strengthen these attitudes.

Table 5 and Supplemental Table S3 show the responses to the practice questions and their relationship to demographic and clinical characteristics, respectively. A total of 544 (44.2%) of the participants always/usually make sure to have important vaccines, while only 340 (27.6%) choose rarely/never. Assurance to take important vaccinations was significantly associated with many demographic and clinical characteristics (*p* < 0.05). Also, a total of 616 (50.1%) of the participants chose always/usually seek medical help when experiencing respiratory symptoms, while only 269 (21.9%) chose rarely/never. Seeking medical help was significantly associated with many demographic characteristics, including age, gender, marital status, nationality, region of residence, employment status, awareness of pneumonia, monthly income, and number of children (*p* ≤ 0.001 each; Table 5). Also, seeking medical help was significantly associated with many clinical characteristics including smoking status, the presence of chronic disease, respiratory condition, chronic kidney disease, history of receiving the pneumococcal vaccine, and whether their children received this vaccines (*p* ≤ 0.001), chronic heart disease (*p* = 0.001) and sickle cell disease (*p* = 0.048; Supplemental Table S3). In addition, a total of 643 (52.3%) of the participants always/usually ask their healthcare providers about the vaccinations they need, while only 238 (19.4%) never/rarely do. This was significantly associated with many demographic and clinical characteristics (*p* < 0.05). Moreover, following doctors’ recommendations regarding vaccination was reported as always/usually by 721 (58.7%) of the participants, while only 225 (18.3%) rarely/never follow such recommendations. This was significantly associated with many demographic and clinical characteristics (*p* < 0.05). Furthermore, a total of 689 (56%) of the participants always/usually attend their healthcare center appointments regularly, while only 241 (19.5%) never/rarely do. Regular attendance at healthcare centers was significantly associated with many demographic and clinical characteristics (*p* < 0.05; Table 5 and Supplemental Table S3).

Importantly, a total of 563 (45.8%) of the participants always/usually encourage family and friends to receive vaccinations, while only a quarter of them (*n* = 301; 24.5%) never/rarely do. This was significantly associated with many demographic and clinical characteristics (*p* < 0.05). To this end, participation in community health events was reported as always/usually by 474 (38.5%) of the participants, while about a third of them (*n* = 414, 33.6%) chose rarely/never participate. This was significantly associated with many demographic and clinical characteristics (*p* < 0.05. Significantly, a total of 630 (51.3%) of the participants reported that they always/usually have time to take their family for vaccinations, while only 272 (22.2%) reported that they rarely/never do. This was significantly associated with many demographic and clinical characteristics (*p* < 0.05). Lastly, 502 (40.8%) of the participants reported that they always/usually ensure that they receive seasonal vaccines, while less than a third of them (*n* = 401; 32.6%) reported that they rarely/never do. The uptake of seasonal vaccines was significantly associated with many demographic and clinical characteristics (*p* < 0.05; Table 5 and Supplemental Table S3).

The average/overall practice score was calculated by taking the average practice score of each participant (the correct practice being given a score of 5 and the improper practice given 1). The average practice score was 68% suggesting a relatively satisfactory level of adherence to recommended vaccination practices among the participants. Statistically significant differences were noted across various practice items in relation to demographic and clinical factors. These findings indicate that while many participants demonstrate positive practices regarding vaccination, efforts are necessary to ensure higher compliance and better health outcomes in the Saudi community.

## 4. Discussion

This study explored the knowledge, attitudes, and practices (KAP) regarding pneumococcal infection and vaccination among Saudi residents. The results revealed that although 77.2% of participants were aware of pneumonia, there was a general lack of knowledge about the pneumococcal disease itself. Despite the high awareness of pneumonia (77.2%), the detailed knowledge about pneumococcal disease itself was low (average knowledge score = 58.6%). This shows a more thorough understanding and differentiation of the disease itself (pneumonia), which has multiple causes, from the particular pneumonia caused by pneumococcal infection. Practices of the participants regarding pneumococcal infection and vaccination were largely above average (68%). Also, despite positive attitudes towards preventive measures (average attitude score = 70.6), a notable 38.3% of the respondents had not received the pneumococcal vaccine, and 40.5% could not recall their vaccination status. Most of the participants were young adults aged 18–30 years, representing 50.2%. The findings also indicated that many sociodemographic factors, such as age, education level, and income, significantly influenced participants’ KAP towards pneumococcal infection and its vaccination. Consequently, the study emphasizes the urgent need for targeted public health campaigns to enhance understanding and increase pneumococcal vaccination rates among diverse demographic groups in Saudi Arabia.

The awareness of pneumococcal vaccination varies significantly across the world [15,23,24,25,26]. In alignment with our findings, one study [10] reported that only 17% of participants were aware of the benefits of pneumococcal vaccination. This suggests a similar trend in public knowledge deficits regarding pneumococcal vaccination in Saudi Arabia. Also, another report [11], emphasized the necessity for enhanced health education campaigns to improve understanding and acceptance of pneumococcal vaccinations. When comparing our results to the broader Middle Eastern contexts, a study in Jordan [27] reported analogous low levels of awareness and acceptance of pneumococcal vaccines. Their findings reinforce the importance of educational initiatives, echoing our recommendations for targeted outreach to improve vaccination uptake in the region. Globally, the landscape of pneumococcal vaccination awareness varies significantly [15,23,24,25,26]. For instance, a study conducted in the United States highlighted higher awareness and uptake rates, attributed to proactive public health campaigns [2]. However, challenges similar to those observed in this study persist in low-income countries, where misinformation and lack of education contribute to low vaccination rates, as reviewed elsewhere [28]. The need for educational interventions to address these knowledge gaps is a common theme across various studies worldwide [29]. Importantly, in our study, the awareness of pneumococcal vaccine inclusion in the national immunization program was low (44.5%), despite the high awareness of vaccination as a preventive measure (88.4%). This may be attributed to the inclusion of the pneumococcal vaccine in the national immunization program in the kingdom only in 2009, as compared to other routine vaccines that were introduced much earlier.

Demographic factors, particularly age and education level, significantly influenced participants’ KAP in our study. In this regard, several studies have shown that sociodemographic factors such as income, employment status and educational level of the individual influence their vaccination behavior [22,30,31,32]. In our study, younger individuals exhibited higher levels of awareness and knowledge about pneumococcal vaccination compared to older participants. This discrepancy may be linked to the integration of health education in school curricula and increased access to information through digital platforms, which younger populations are more likely to utilize [33]. Conversely, older age groups may have limited exposure to these resources, indicating a need for targeted outreach efforts that specifically address their concerns and provide them with relevant information [33]. This is reflected in the low vaccination coverage of older adults (only 12.9%) [14]. Also, although the Saudi Ministry of Health reports show that the annual pneumococcal vaccination coverage among Saudi children hovered around 98%, about 29.9% of the participants reported that their children had not received the pneumococcal vaccine. Moreover, only 44.2% of the participants said that they always/usually make sure their children and family members have the important vaccinations in the Kingdom, including the pneumococcal vaccine. This suggests either a recall bias or that the participant him/herself does not closely follow their children’s vaccination status.

The attitudes of our participants towards pneumococcal vaccination were generally positive (Average attitude score = 70.6%), with many acknowledging its importance in preventing serious health complications of pneumococcal infections. This aligns with local findings. For example, one report [9] showed that 50.6% of participants agreed on the safety of vaccines, though some expressed concerns about the pneumococcal vaccine specifically. Regionally, a study in Jordan [27] found that while participants recognized the importance of vaccination, they voiced apprehensions about vaccine safety, indicating a similar trend in public attitudes in the region. On the other hand, in the United States, one study [2] emphasized that effective public health campaigns have fostered significantly higher awareness and acceptance of pneumococcal vaccination, showcasing a clearer distinction when contrasted with the findings in Saudi Arabia.

In terms of practices related to pneumococcal vaccination, this study found that many of the participants did not follow recommended vaccination schedules. The mean practice score of our participants was above average (68%). A considerable number of the participants indicated that they either had not received the vaccine or could not recall their vaccination status. This points to a critical gap in vaccination adherence, which aligns with findings from regional studies. For instance, one report [9] noted that only 30% of participants in their study reported receiving the pneumococcal vaccine on schedule. Similarly, in the United Arab Emirates, a study found that while the awareness levels were relatively high, actual vaccination rates were low due to misconceptions and a lack of reminders from healthcare providers [15]. Globally, studies indicate that, particularly in low-income countries, similar challenges persist where cultural beliefs and lack of health education contribute to low vaccination rates, as reviewed elsewhere [28]. To improve adherence rates, it is essential to implement systematic reminders and follow-up mechanisms that encourage individuals to stay up-to-date with their vaccinations.

Based on our findings, several recommendations emerge for enhancing KAP regarding pneumococcal infection and vaccination among Saudi residents. First, targeted public health campaigns should focus on improving awareness about pneumococcal infections and the importance of its vaccination. These campaigns can utilize various platforms, including social media, community workshops, and collaboration with local healthcare providers to reach diverse audiences. We speculate that a collective effort through social media, religious leaders, SMS and healthcare workers at both hospitals and clinics can reduce the identified gaps in selected groups, such as those with low socioeconomic status and low education levels. Second, as many citizens of the kingdom do not know Arabic very well, instructional materials should be created in a variety of languages and at different literacy levels to enable accessibility for various demographic groups. Finally, engaging visual aids and interactive content can further enhance audience engagement. Moreover, training healthcare providers in effective communication strategies is essential to address patients’ concerns about vaccine safety and efficacy. This training can foster a more trusting relationship between healthcare providers and patients, encouraging individuals to seek vaccinations. Community engagement is also crucial and involving community leaders and influencers in vaccination campaigns can enhance credibility and outreach. Additionally, community-based programs can help dispel myths and encourage discussions about the benefits of pneumococcal vaccination.

This study’s strengths include a substantial sample size and a focus on diverse demographic groups, participation of large numbers of participants from the different sociodemographic groups and different regions of the kingdom, which supports the generalization of the findings. However, this study has several limitations, including potential recall bias, as participants may have provided socially desirable answers regarding their knowledge, attitudes, and practices. Additionally, a cross-sectional design limits the ability to establish causal relationships between demographic factors and KAP outcomes. Consequently, the generalization of our findings should be interpreted with caution. Future research should consider longitudinal studies to better understand the dynamics of KAP over time and the effectiveness of targeted interventions.

## 5. Conclusions

In conclusion, this study underscores the critical need for improved knowledge, attitudes, and practices regarding pneumococcal infection and vaccination among Saudi residents. By addressing the identified gaps through targeted educational initiatives and community engagement, public health authorities can enhance pneumococcal vaccine uptake and ultimately reduce the burden of pneumococcal diseases among the Saudi population. Further research is warranted to explore the long-term effectiveness of these interventions and to continuously assess the population’s KAP regarding pneumococcal vaccination.

## Figures and Tables

**Table 1:** Demographic characteristics of the study participants.

Question	Category	Frequency (%)
Gender	Female	630 (51.2)
	Male	600 (48.8)
Age	18–30	617 (50.2)
	31–40	231 (18.8)
	41–50	216 (17.6)
	51–60	128 (10.4)
	61 and above	38 (3.1)
Marital status	Single	569 (46.3)
	Married	498 (40.5)
	Divorced	116 (9.4)
	Widowed	47 (3.8)
Nationality	Saudi	1075 (87.4)
	Non-Saudi	155 (12.6)
Region of Residence	Southern region	156 (12.7)
	Eastern region	126 (10.2)
	Northern region	120 (9.8)
	Western region	689 (56.0)
	Central region	139 (11.3)
Employment status	Unemployed/student/private business	624 (50.7)
	Employee at private sector	123 (10.0)
	Healthcare worker in government sector; including military sector	115 (9.3)
	Healthcare worker (private sector)	124 (10.1)
	Employee at another governmental sector	224 (19.8)
Education level	Bachelor’s degree	719 (58.5)
	Doctorate (PhD)	28 (2.3)
	Master’s degree	93 (7.6)
	High school diploma or less	390 (31.7)
Monthly income	<5000 SAR	693 (56.3)
	5000–10,000 SAR	357 (29.0)
	>10,000 SAR	180 (14.6)
Have you ever heard of pneumonia	Yes	950 (77.2)
	No	280 (22.8)
Source of information: - mass media (TV and newspapers)	Yes	589 (47.9)
	No	641 (52.1)
- social media	Yes	734 (59.7)
	No	496 (40.3)
- Family, friends, and colleagues	Yes	588 (47.8)
	No	642 (52.2)
- Books and magazines	Yes	357 (29.0)
	No	873 (71.0)
- Academic studies	Yes	539 (43.8)
	No	691 (56.2)
Number of children	More than three	162 (13.2)
	Three	134 (10.9)
	Two	233 (18.9)
	One	90 (7.3)
	Zero	611 (49.7)

**Table 2:** Clinical characteristics of the study participants.

Question	Category	Frequency (%)
Smoking status	Non-smoker	920 (74.8)
	Smoker	206 (16.7)
	Former smoker	104 (8.5)
Chronic illness	Yes	350 (28.5)
	No	880 (71.5)
Respiratory conditions (asthma, COPD)	Yes	288 (23.4)
	No	942 (76.6)
Chronic heart disease (including hypertension)	Yes	176 (14.3)
	No	1054(85.7)
Chronic kidney disease	Yes	163 (13.3)
	No	1067 (86.7)
Sickle cell disease	Yes	139 (11.3)
	No	1091 (88.7)
Any allergies	Yes	243 (19.8)
	No	987 (80.2)
Other	Yes	217 (17.6)
	No	1013 (82.4)
Received pneumococcal vaccine	No	477 (38.8)
	Yes	255 (20.7)
	I do not remember	498 (40.5)
Have your children receive pneumococcal vaccine	No	368 (29.9)
	I don’t remember	212 (17.2)
	Yes, some of them	139 (11.3)
	Yes, all of them	134 (10.9)
	Not applicable	377 (30.7)

**Table 3:** Distribution of responses to knowledge questions and its correlation with demographic characteristics.

Question	Category	F (%) *	KS (%) *	A *	G *	MS *		N *	RA *	ES *	EL *	MI *	Ever Heard of Pneumonia	NC *
What causes pneumococcal infection?	Bacteria	487 (39.6)	**39.6**	**<0.001**	0.670	**<0.001**		**<0.001**	**<0.001**	**<0.001**	**<0.001**	**<0.001**	**<0.001**	**<0.001**
	Virus	302 (24.6)												
	Fungi	57 (4.6)												
	I do not know	384 (31.2)												
How is pneumococcal infection transmitted?														
- air (coughing, sneezing)	Yes	1041 (84.6)	**84.6**	**<0.001**	**0.002**	**<0.001**		0.603	0.190	**0.001**	0.439	**0.010**	**<0.001**	**0.003**
	No	189 (15.4)												
- contaminated water or food	Yes	562 (45.7)	**54.3**	0.554	0.482	0.186		0.471	0.124	0.115	**0.014**	0.925	**0.014**	0.620
	No	668 (54.3)												
- direct physical contact	Yes	565 (45.9)	**45.9**	**0.049**	0.192	0.257		0.890	0.168	0.064	0.887	0.511	**0.002**	0.255
	No	665 (54.1)												
What are the common symptoms of Pneumococcal infection?														
- Fever	Yes	958 (77.9)	**77.9**	**<0.001**	**0.017**	**<0.001**		**0.003**	0.093	**<0.001**	0.050	**<0.001**	**<0.001**	**<0.001**
	No	272 (22.1)												
- Vomiting	Yes	605 (49.2)	**50.8**	**0.002**	0.113	**<0.001**		**0.007**	**<0.001**	**<0.001**	**0.002**	**0.002**	**0.045**	**<0.001**
	No	625 (50.8)												
- Cough	Yes	827 (67.2)	**67.2**	**<0.001**	**<0.001**	**<0.001**		**<0.001**	**<0.001**	**<0.001**	**<0.001**	**<0.001**	**<0.001**	**<0.001**
	No	403 (32.8)												
- Fatigue, and rapid or difficulty breathing	Yes	812 (66)	**66.0**	**<0.001**	**<0.001**	**<0.001**		**<0.001**	**<0.001**	**<0.001**	0.567	0.344	**<0.001**	**<0.001**
	No	418 (34)												
Who is at high-risk of pneumococcal infection?														
- Infants and young children	Yes	970 (78.9)	**78.9**	**<0.001**	0.316	**<0.001**		**0.002**	**<0.001**	**<0.001**	**0.009**	**0.001**	**<0.001**	**<0.001**
	No	260 (21.1)												
- Pregnant women	Yes	722 (58.7)	**41.3**	**<0.001**	0.158	**<0.001**		**0.002**	**<0.001**	**<0.001**	0.252	**<0.001**	**0.003**	**<0.001**
	No	508 (41.3)												
- Adults less than 65 years	Yes	429 (34.9)	**65.1**	**0.004**	0.744	**0.007**		0.211	0.617	0.178	0.182	0.256	**0.036**	0.181
	No	801 (65.1)												
- Adults over 65 years old	Yes	782 (63.6)	**63.6**	**<0.001**	0.140	**<0.001**		**0.002**	**<0.001**	**<0.001**	**0.404**	0.146	**<0.001**	**<0.001**
	No	448 (36.4)												
- Those with weakened immune systems	Yes	951 (77.3)	**77.3**	**<0.001**	**<0.001**	**<0.001**		**0.008**	**<0.001**	**<0.001**	**<0.001**	**<0.001**	**<0.001**	**<0.001**
	No	279 (22.7)												
Pneumococcal infection leads to serious health complication	Yes	639 (52)	**52.0**	**<0.001**	**<0.001**	**<0.001**		**<0.001**	**<0.001**	**<0.001**	**0.001**	**0.003**	**<0.001**	**<0.001**
	No	174 (14.1)												
	I don’t know	417 (33.9)												
How can person prevent getting pneumococcal infection?														
- Vaccination	Yes	1087 (88.4)	**88.4**	0.178	**<0.001**	0.162		0.793	**<0.001**	**0.038**	**0.048**	**0.039**	**<0.001**	**<0.001**
	No	143 (11.6)												
- Avoid eating at restaurants	Yes	569 (46.3)	**53.7**	**<0.001**	**0.001**	**<0.001**		<0.001	**<0.001**	<0.001	**<0.001**	**0.002**	0.841	**<0.001**
	No	661 (53.7)												
- Covering mouth and nose when coughing or sneezing	Yes	863 (70.2)	**70.2**	**<0.001**	**<0.001**	**<0.001**		<0.001	**<0.001**	**<0.001**	**<0.001**	**<0.001**	**<0.001**	**<0.001**
	No	367 (29.8)												
- Avoid shaking hands	Yes	592 (48.1)	**48.1**	**0.016**	0.980	0.549		0.655	0.321	0.996	0.754	0.123	0.082	0.077
	No	638 (51.9)												
A patient with pneumococcal infection can be cured	Yes	628 (51.1)	**51.1**	**<0.001**	**0.009**	**<0.001**		**<0.001**	**<0.001**	**<0.001**	**0.003**	**<0.001**	**<0.001**	**<0.001**
	No	144 (11.7)												
	I don’t know	458 (37.2)												
There is a vaccine for pneumococcal infection	Yes	637 (51.8)	**51.8**	**<0.001**	**<0.001**	**<0.001**		**<0.001**	**<0.001**	**<0.001**	**<0.001**	**<0.001**	**<0.001**	**<0.001**
	No	163 (13.3)												
	I don’t know	430 (35)												
pneumococcal vaccine is a part of the national immunization schedule	Yes	547 (44.5)	**44.5**	**<0.001**	**<0.001**	**<0.001**		**<0.001**	**<0.001**	**<0.001**	**<0.001**	**<0.001**	**<0.001**	**<0.001**
	No	143 (11.6)												
	I don’t know	540 (43.9)												
How many doses of the pneumococcal vaccine are needed	Two doses	812 (66)	**25.4**	**<0.001**	**0.631**	**<0.001**		**0.005**	**<0.001**	**<0.001**	0.059	**<0.001**	0.887	**<0.001**
	Four doses	312 (25.4)												
	Six doses	106 (8.6)												
How effective is the pneumococcal vaccine ?	Very effective	406 (33)	**73.9**	**0.004**	**0.014**	**<0.001**		0.138	0.137	**0.008**	0.521	0.179	**<0.001**	**<0.001**
	effective	503 (40.9)												
	Somewhat effective	270 (22)												
	Not effective	51 (4.1)												
The flu vaccine is the same as pneumococcal vaccine	Yes	309 (25.1)	**35.7**	**<0.001**	**<0.001**	**<0.001**		**<0.001**	**<0.001**	**<0.001**	**<0.001**	**<0.001**	**<0.001**	**<0.01**
	No	439 (35.7)												
	I don’t know	482 (39.2)												
Average knowledge score							**58.6**							

* F, Frequency KS, Knowledge score A, Age G, Gender MS, Marital status N, Nationality RA, Region of residence ES, Employment Status El, Education level MI, Monthly Income NC, Number of children.

**Table 4:** Distribution of responses to attitude questions and its correlation with demographic characteristics.

Statements	Response N (%)						*p*-Value									
	* SA	* A	* N	* D	* SD	Attitude Score	Age	Gender	** MS	** N	** RA	** ES	** EL	** MI	Heard of Pneumonia	** NC
Pneumococcal infection is a serious health threat.	171 (13.9)	426 (34.6)	316 (25.7)	189 (15.4)	128 (10.4)	**65.3**	**<0.001**	**<0.001**	**<0.001**	**<0.001**	**<0.001**	**<0.001**	**<0.001**	**<0.001**	**0.001**	**<0.001**
It is important for individuals at risk to receive the pneumococcal vaccine.	341 (27.7)	407 (33.1)	277 (22.5)	114 (9.3)	91 (7.4)	**72.9**	**<0.001**	**0.001**	**<0.001**	**<0.001**	**<0.001**	**<0.001**	**0.005**	**<0.001**	**0.002**	**<0.001**
The pneumococcal vaccine is effective in preventing pneumonia infection.	247 (20.1)	498 (40.5)	325 (26.4)	91 (7.4)	69 (5.6)	**72.4**	**<0.001**	**<0.001**	**<0.001**	**0.043**	**<0.001**	**<0.001**	0.698	0.050	**<0.001**	**<0.001**
When people get pneumococcal vaccine, it can help limiting the spread of pneumococcal infection.	313 (25.4)	469 (38.1)	270 (22)	101 (8.2)	77 (6.3)	**73.7**	**<0.001**	**<0.001**	**<0.001**	**0.009**	**<0.001**	**<0.001**	0.875	0.128	**<0.001**	**<0.001**
I must recommend the pneumococcal vaccine to my family and friends.	293 (23.8)	459 (37.3)	308 (25)	101 (8.2)	69 (5.6)	**73.1**	**<0.001**	**<0.001**	**<0.001**	**<0.001**	**<0.001**	**<0.001**	**0.004**	**0.004**	**<0.001**	**<0.001**
It is important for healthcare workers to receive the pneumococcal vaccine.	377 (30.7)	433 (35.2)	264 (21.5)	92 (7.5)	64 (5.2)	**75.7**	**<0.001**	**<0.001**	**<0.001**	**<0.001**	**<0.001**	**<0.001**	**<0.001**	**<0.001**	**<0.001**	**<0.001**
I am concerned about the possible side effects of the pneumococcal vaccine.	208 (16.9)	400 (32.5)	396 (32.2)	144 (11.7)	82 (6.7)	**68.3**	**<0.001**	**0.010**	**<0.001**	0.120	**<0.001**	**0.002**	0.458	**0.001**	0.531	**<0.001**
I follow the medical advice to get the pneumococcal vaccine if recommended by a healthcare professional.	327 (26.6)	466 (37.9)	248 (20.2)	103 (8.4)	86 (7)	**73.7**	**<0.001**	**<0.001**	**<0.001**	**<0.001**	**<0.001**	**<0.001**	**0.006**	**0.004**	**<0.001**	**<0.001**
My family and children are healthy and does not need pneumococcal vaccination.	122 (9.9)	310 (25.2)	401 (32.6)	239 (19.4)	158 (12.8)	**60**	**<0.001**	**0.117**	**<0.001**	**0.033**	**0.001**	**0.001**	**0.080**	**0.002**	**0.005**	**<0.001**
Average attitude score	**70.56**															

* SD, strongly disagree. D, Disagree. N, Neutral. A, Agree. SA, strongly agree ** MS, Marital status N, Nationality RA, Region of residence ES, Employment Status El, Education level MI, Monthly Income NC, Number of children.

**Table 5:** Distribution of responses to practice questions and their correlation with demographic characteristics.

Statements	Always N (%)	Usually N (%)	Sometimes N (%)	Rarely N (%)	Never N (%)	Practice Score	Age	Gender	** MS	** N	** RA	** ES	** EL	** MI	Have You Heard of Pneumonia	** NC
I make sure my children and family members get the important vaccinations in the Kingdom, including the pneumococcal vaccine.	305 (24.8)	239 (19.4)	346 (28.1)	222 (18)	118 (9.6)	**66.4**	**<0.001**	**0.001**	**<** **0.001**	**<0.001**	**<0.001**	**<0.001**	0.218	**<0.001**	**<0.001**	**<0.001**
I seek medical help when I experience symptoms of pneumococcal infection (fever, headache, nasal congestion, chest pain).	320 (26)	296 (24.1)	345 (28)	146 (11.9)	123 (10)	**68.8**	**<0.001**	**<0.001**	**<0.001**	**<0.001**	**<0.001**	**<0.001**	0.052	**0.001**	**<0.001**	**<0.001**
I ask my healthcare providers about the vaccination I need.	387 (31.5)	256 (20.8)	349 (28.4)	136 (11.1)	102 (8.3)	**71.2**	**0.006**	**0.100**	**0.010**	0.081	**0.022**	0.154	0.562	0.144	**<0.001**	**0.020**
I follow my doctor’s recommendations regarding vaccination.	457 (37.2)	264 (21.5)	284 (23.1)	119 (9.7)	106 (8.6)	**73.8**	**<0.001**	**<0.001**	**<0.001**	**<0.001**	**<0.001**	**<0.001**	**0.002**	**0.002**	**<0.001**	**<0.001**
I regularly attend my appointments at the primary healthcare center.	418 (34)	271 (22)	300 (24.4)	142 (11.5)	99 (8)	**72.5**	**<0.001**	**<0.001**	**<0.001**	**<0.001**	**<0.001**	**<0.001**	0.097	**0.001**	**0.009**	**<0.001**
I encourage my friends and my family to get vaccinated against pneumococcal disease.	296 (24.1)	267 (21.7)	366 (29.8)	152 (12.4)	149 (12.1)	**66.7**	**<0.001**	0.102	**<0.001**	**0.016**	**0.001**	**<0.001**	0.485	**0.001**	**<0.001**	**0.001**
I participate in community health events or programs that promote vaccination.	250 (20.3)	224 (18.2)	342 (27.8)	196 (15.9)	218 (17.7)	**61.5**	0.244	0.192	0.100	**0.012**	**0.019**	**0.014**	0.131	**0.014**	0.344	**0.006**
I have time to take my family and children for vaccination.	318 (25.9)	312 (25.4)	328 (26.7)	147 (12)	125 (10.2)	**69**	**<0.001**	0.601	**0.002**	0.299	0.056	**0.001**	**0.032**	**<0.001**	0.197	**0.003**
I make sure to get seasonal vaccines.	239 (19.4)	263 (21.4)	327 (26.6)	208 (16.9)	193 (15.7)	**62.4**	**0.002**	0.701	**0.008**	**0.012**	**<0.001**	**0.004**	**0.017**	**0.029**	**0.015**	**0.023**
**Average practice score:**							**68**									

** MS, Marital status N, Nationality RA, Region of residence ES, Employment Status El, Education level MI, Monthly Income NC, Number of children.

## Data Availability

The data sets used and/or analyzed during this study are available from the corresponding author on reasonable request.

## Supplementary Materials

The following supporting information can be downloaded at: https://www.mdpi.com/article/10.3390/pathogens14070711/s1, Supplemental Table S1. Distribution of responses to knowledge questions and its correlation with clinical characteristics, Supplemental Table S2. Distribution of responses to attitude questions and its correlation with clinical characteristics, Supplemental Table S3. Distribution of responses to practice questions and its correlation with clinical characteristics.
