# Supplementary material for: Pneumococcal Infection and Vaccination-Related Knowledge, Attitudes and Practices Among Saudi Residents
Source: Pathogens. 2025 Jul 18;14(7):711. doi: 10.3390/pathogens14070711 (PMC12299447; doi:10.3390/pathogens14070711)
Supplement: Supplementary file 1 [file pathogens-14-00711-s001.zip › pathogens-3717109-supplementary.pdf]

Article

Pneumococcal Infection and Vaccination-Related Knowledge, Attitudes and Practices Among Saudi Residents

Wed S. Althobaiti [1], Abeer D. Alnefaie [1], Kaifah M. Althaali [1], Ola M. Alsufyani [1], Yassmin M. Shebany [2], Ayman A. Atalla [3], Ibtihal M. Alotaibi [1], Eman Y. Santali [4] and Sayed F. Abdelwahab [5]

[1] College of Pharmacy, Taif University, Taif 21944, Saudi Arabia
[2] Biology Department, Faculty of Science, Taif University, Taif 21944, Saudi Arabia
[3] Family Medicine Department, College of Medicine, Taif University, Taif 21944
[4] Pharmaceutical Chemistry Department, College of Pharmacy, Taif University, Taif 21944, Saudi Arabia
[5] Pharmaceutics and Industrial Pharmacy Department, College of Pharmacy, Taif University, Taif 21944, Saudi Arabia.
* Correspondence: s.fekry@tu.edu.sa; **ORCID**: 0000-0002-9636-7485

Supplemental Table S1. Distribution of responses to knowledge questions and its correlation with clinical characteristics.

Questions	Response N (%)			P- value									
	Category	F (%)*	KS (%)*	Smoking Status	Chronic illnesses	RC*	CHD*	CKD*	Sickle Cell disease	Any Allergies	Others	RPV*	CRPV*
What causes pneumococcal infection?	Bacteria	487 (39.6)	**39.6**	**<0.001**	**<0.001**	**<0.001**	**<0.001**	**<0.001**	**<0.001**	**<0.001**	**<0.001**	**<0.001**	**<0.001**
	Virus	302 (24.6)											
	Fungi	57 (4.6)											
	I don’t know	384 (31.2)											
**How is pneumococcal infection transmitted?**													
Through air (coughing, sneezing)	Yes	1041 (84.6)	**84.6**	0.947	**0.016**	**<0.001**	**0.001**	**<0.001**	**0.001**	**0.010**	**0.004**	0.060	**0.028**
	No	189 (15.4)											
	Yes	562 (45.7)	**54.3**	0.382	0.208	0.229	0.715	0.799	0.788				

Through contaminated water or food	No	668 (54.3)								0.841	0.403	0.240	0.167
	Yes	565 (45.9)	**45.9**	0.307	0.121	**0.020**	0.771	**0.005**	0.508	0.876	**0.006**	0.283	0.057
-Through direct physical contact	No	665 (54.1)											
**What are the common symptoms of Pneumococcal infection?**													
- Fever	Yes	958 (77.9)	**77.9**	0.148	**<0.001**	**<0.001**	**<0.001**	**<0.001**	**<0.001**	**<0.001**	**<0.001**	**<0.001**	**<0.001**
	No	272 (22.1)											
- Vomiting	Yes	605 (49.2)	**50.8**	<0.001	**<0.001**	**<0.001**	**<0.001**	**<0.001**	**0.004**	**0.005**	**0.001**	**<0.001**	**<0.001**
	No	625 (50.8)											
- Cough	Yes	827 (67.2)	**67.2**	**<0.001**	**<0.001**	**<0.001**	**<0.001**	**<0.001**	**<0.001**	**<0.001**	**<0.001**	**<0.001**	**<0.001**
	No	403 (32.8)											
- Fatigue, and rapid or difficulty breathing	Yes	812 (66)	**66.0**	**<0.001**	**<0.001**	**<0.001**	**<0.001**	**0.002**	**0.007**	0.054	0.075	**<0.001**	**<0.001**
	No	418 (34)											
**Who is at the high risk of pneumococcal infection?**													
- Infants and young children	Yes	970 (78.9)	**78.9**	0.084	**0.002**	**<0.001**	**0.001**	**<0.001**	**0.002**	**0.022**	**0.004**	**<0.001**	**<0.001**
	No	260 (21.1)											
- Pregnant women	Yes	722 (58.7)	**41.3**	0.033	0.107	**0.005**	**0.001**	0.834	0.720	0.251	0.158	**0.047**	**<0.001**
	No	508 (41.3)											
- Adults less than 65 years old	Yes	429 (34.9)	65.1	0.338	**0.048**	0.159	0.581	0.064	0.127	0.425	0.517	**0.002**	**0.001**
	No	801 (65.1)											
- Adults over 65 years old	Yes	782 (63.6)	63.6	**<0.001**	**<0.001**	**<0.001**	**<0.001**	**0.004**	**0.005**	**0.013**	0.096	**<0.001**	**<0.001**
	No	448 (36.4)											
-Those with weakened immune systems	Yes	951(77.3)	**77.3**	**<0.001**	**<0.001**	**<0.001**	**<0.001**	**<0.001**	**<0.001**	**0.001**	**<0.001**	**<0.001**	**<0.001**
	No	279 (22.7)											
	Yes	639 (52.0)	**52.0**	**<0.001**	**<0.001**	**<0.001**	**<0.001**	**<0.001**	**<0.001**	**0.006**	**0.001**	**<0.001**	**<0.001**

Pneumococcal infection leads to serious health complications	No	174 (14.1)											
	I don't know	417 (33.9)											
**How can person prevent getting pneumococcal infection?**													
-Vaccination	Yes	1087 (88.4)	**88.4**	0.147	**0.005**	**<0.001**	**<0.001**	**<0.001**	**<0.001**	**<0.001**	**<0.001**	0.119	**<0.001**
	No	143 (11.6)											
-Avoid eating at restaurants	Yes	569 (46.3)	**53.7**	**<0.001**	**<0.001**	**<0.001**	**<0.001**	**<0.001**	**<0.001**	**0.005**	**<0.001**	**<0.001**	**<0.001**
	No	661 (53.7)											
-Covering mouth and nose when coughing or sneezing	Yes	863 (70.2)	**70.2**	**<0.001**	**<0.001**	**<0.001**	**<0.001**	**<0.001**	**<.0.001**	**<0.001**	**<0.001**	**<0.001**	**<0.001**
	No	367 (29.8)											
- Avoid shaking hands	Yes	592 (48.1)	**48.1**	0.698	0.654	0.410	0.756	0.780	0.182	0.782	0.122	0.433	**0.004**
	No	638 (51.9)											
**A patient with pneumococcal infection can be cured**	Yes	628 (51.1)	**51.1**	**<0.001**	**<0.001**	**<0.001**	**<0.001**	**<0.001**	**<0.001**	**<0.001**	**<0.001**	**<0.001**	**<0.001**
	No	144 (11.7)											
	I don't know	458 (37.2)											
There is a vaccine available for pneumococcal infection.	Yes	637 (51.8)	**51.8**	**<0.001**	**<0.001**	**<0.001**	**<0.001**	**<0.001**	**<0.001**	**<0.001**	**0.001**	**<0.001**	**<0.001**
	No	163 (13.3)											
	I don't know	430 (35)											
pneumococcal vaccine is a part of the national immunization	Yes	547 (44.5)	**44.5**	**<0.001**	**<0.001**	**<0.001**	**<0.001**	**<0.001**	**<0.001**	**<0.001**	**<0.001**	**<0.001**	**<0.001**
	No	143 (11.6)											
	I don't know	540 (43.9)											
	Two doses	812 (66)	**25.4**	**<0.001**	**<0.001**	**0.001**	**0.011**	**0.033**	**0.001**	0.097	**0.016**	**<0.001**	**<0.001**
	Four doses	312 (25.4)											

**How many doses of the pneumococcal vaccine are needed**	Six doses	106 (8.6)											
**How effective do you think is the pneumococcal vaccine in preventing infection?**	Very effective	406 (33)	**73.9**	0.843	**0.018**	0.074	0.092	0.052	0.210	0.154	0.657	**0.007**	**<0.001**
	effective	503 (40.9)											
	Somewhat effective	270 (22)											
	Not effective	51 (4.1)											
**The flu vaccine is the same as pneumococcal vaccine**	Yes	309 (25.1)	**35.7**	**<0.001**	**<0.001**	**<0.001**	**<0.001**	**<0.001**	**<0.001**	**<0.001**	**<0.001**	**<0.001**	**<0.001**
	No	439 (35.7)											
	I don't know	482 (39.2)											
**Average knowledge score**			**58.6**										

***F**, Frequency. **KS**, Knowledge score. **RC**, Respiratory conditions. **CHD**, Chronic heart disease. **CKD**, Chronic kidney disease. **RPV**, Received pneumococcal vaccine. **CRPV**, children received pneumococcal vaccine.

Supplemental Table S2. Distribution of responses to attitude questions and its correlation with clinical characteristics.

Statements	Response N (%)						P-value									
	*SA	*A	*N	*D	*SD	Attitude score	Smoking status	Chronic disease	Respiratory conditions	**CHD	**CKD	**SCD	Allergy	Other	**RV	**CRV
Pneumococcal infection is a serious health threat.	171 (13.9)	426 (34.6)	316 (25.7)	189 (15.4)	128 (10.4)	**65.3**	**<0.001**	**<0.001**	**<0.001**	**<0.001**	**<0.001**	**<0.001**	**<0.001**	**<0.001**	**<0.001**	**<0.001**
It is important for individuals at risk e.g., children, adults over 65 years and those with weakened immune system to receive the pneumococcal vaccine.	341 (27.7)	407 (33.1)	277 (22.5)	114 (9.3)	91 (7.4)	**72.9**	**<0.001**	**<0.001**	**<0.001**	**<0.001**	**<0.001**	**<0.001**	**0.001**	**<0.001**	**<0.001**	**<0.001**
The pneumococcal vaccine is effective in preventing pneumonia infection.	247 (20.1)	498 (40.5)	325 (26.4)	91 (7.4)	69 (5.6)	**72.4**	**<0.001**	**<0.001**	**<0.001**	**<0.001**	**<0.001**	**<0.001**	**0.007**	**0.008**	**<0.001**	**<0.001**

When people get pneumococcal vaccine, it can help limiting the spread of pneumococcal infection.	313 (25.4)	469 (38.1)	270 (22)	101 (8.2)	77 (6.3)	**73.7**	**<0.001**	**<0.001**	**<0.001**	**<0.001**	**0.014**	**0.001**	**0.025**	**0.017**	**<0.001**	**<0.001**
I must recommend the pneumococcal vaccine to my family and friends.	293 (23.8)	459 (37.3)	308 (25)	101 (8.2)	69 (5.6)	**73.1**	**<0.001**	**<0.001**	**<0.001**	**<0.001**	**<0.001**	0.198	**0.049**	**0.002**	**<0.001**	**<0.001**
It is important for healthcare workers to receive the pneumococcal vaccine.	377 (30.7)	433 (35.2)	264 (21.5)	92 (7.5)	64 (5.2)	**75.7**	**<0.001**	**<0.001**	**<0.001**	**<0.001**	**<0.001**	**<0.001**	**0.001**	**0.013**	**<0.001**	**<0.001**
I am concerned about the possible side effects of the pneumococcal vaccine.	208 (16.9)	400 (32.5)	396 (32.2)	144 (11.7)	82 (6.7)	**68.3**	**<0.001**	**0.034**	**0.017**	**<0.001**	**0.049**	**0.007**	**0.027**	0.143	**0.035**	**<0.001**
I follow the medical advice to get the pneumococcal vaccine if recommended by a healthcare professional.	327 (26.6)	466 (37.9)	248 (20.2)	103 (8.4)	86 (7)	**73.7**	**<0.001**	**<0.001**	**<0.001**	**<0.001**	**<0.001**	**<0.001**	**0.013**	0.060	**<0.001**	**<0.001**

My family and children are healthy and does not need pneumococcal vaccination.	122 (9.9)	310 (25.2)	401 (32.6)	239 (19.4)	158 (12.8)	**60**	0.090	**0.002**	**0.014**	**0.003**	**0.007**	**0.028**	0.466	0.065	**<0.001**	**<0.001**
**Average attitude score**	**70.56**															

* SD, strongly disagree. D, Disagree. N, Neutral. A, Agree. SA, strongly agree . ** CHD, Chronic heart disease CKD, chronic kidney disease SCD, Sickle cell disease RV, Receive pneumococcal vaccine CRV, have your children receive pneumococcal vaccine

Supplemental Table S3. Distribution of responses to practice questions and its correlation with clinical characteristics.

Statements	Always N (%)	Usually N (%)	Sometimes N (%)	Rarely N (%)	Never N (%)	**Practice score**	Smoking status	Chronic disease	Respiratory conditions	**CHD	**CKD	**SCD	Allergy	Other	**RV	**CRV
I make sure my children and family members get the important vaccinations in the Kingdom, including the pneumococcal vaccine.	305 (24.8)	239 (19.4)	346 (28.1)	222 (18)	118 (9.6)	**66.4**	**<0.001**	**<0.001**	**<0.001**	**<0.001**	**<0.001**	**0.002**	**0.006**	**<0.001**	**<0.001**	**<0.001**
I seek medical help when I experience symptoms of pneumococcal infection (fever, headache, nasal congestion, chest pain).	320 (26)	296 (24,1)	345 (28)	146 (11.9)	123 (10)	**68.8**	**<0.001**	**<0.001**	**<0.001**	**0.001**	**<0.001**	**0.048**	0.483	0.069	**<0.001**	**<0.001**
I ask my healthcare providers about the vaccination I need.	387 (31.5)	256 (20.8)	349 (28.4)	136 (11.1)	102 (8.3)	**71.2**	0.087	0.978	0.052	**0.005**	0.216	0.379	0.172	0.147	0.122	**<0.001**
I follow my doctor's recommendations regarding vaccination.	457 (37.2)	264 (21.5)	284 (23.1)	119 (9.7)	106 (8.6)	**73.8**	**<0.001**	**<0.001**	**0.001**	**<0.001**	**0.006**	**0.003**	0.092	0.089	**<0.001**	**<0.001**
I regularly attend my appointments at the primary healthcare center.	418 (34)	271 (22)	300 (24.4)	142 (11.5)	99 (8)	**72.5**	**<0.001**	**<0.001**	**<0.001**	**<0.001**	**0.001**	0.006	**<0.001**	**<0.001**	**<0.001**	**<0.001**

I encourage my friends and my family to get vaccinated against pneumococcal disease.	296 (24.1)	267 (21.7)	366 (29.8)	152 (12.4)	149 (12.1)	**66.7**	**0.011**	**0.002**	0.065	**<0.001**	0.108	0.050	0.383	**0.002**	0.359	**<0.001**
I participate in community health events or programs that promote vaccination.	250 (20.3)	224 (18.2)	342 (27.8)	196 (15.9)	218 (17.7)	**61.5**	0.327	**<0.001**	**<0.001**	**<0.001**	**<0.001**	**<0.001**	**<0.001**	**<0.001**	**0.001**	**0.006**
I have time to take my family and children for vaccination.	318 (25.9)	312 (25.4)	328 (26.7)	147 (12)	125 (10.2)	**69**	0.065	**0.019**	**0.012**	0.086	**0.019**	**0.008**	0.424	**0.010**	**0.004**	**<0.001**
I make sure to get seasonal vaccines.	239 (19.4)	263 (21.4)	327 (26.6)	208 (16.9)	193 (15.7)	**62.4**	0.628	**0.010**	**0.015**	0.136	**<0.001**	**0.002**	0.445	**0.002**	**0.001**	**0.015**
**Average Practice score**							**68**									

**CHD, Chronic heart disease CKD, chronic kidney disease SCD, Sickle cell disease RV, Receive pneumococcal vaccine CRV, have your children receive pneumococcal vaccine.
